# Long-acting reversible and permanent contraceptives utilization and its associated factors among married women who desire no more children in Ethiopia: A multilevel analysis

**DOI:** 10.1371/journal.pone.0316799

**Published:** 2025-01-24

**Authors:** Nuhamin Tesfa Tsega, Wondimnew Mersha Biset, Getie Mihret Aragaw, Saron Abeje Abiy, Tilahun Nega Godana, Abera Dessie Dagnaw, Gashaw Awoke Haile, Daniel Gashaneh Belay, Berihun Agegn Mengistie

**Affiliations:** 1 Department of Women’s and Family Health, School of Midwifery, College of Medicine and Health Sciences, University of Gondar, Gondar, Ethiopia; 2 Department of Anesthesiology, Critical Care and Pain Medicine, Saint Paul’s Hospital Millennium Medical College, Addis Ababa, Ethiopia; 3 Department of General Midwifery, School of Midwifery, College of Medicine and Health Sciences, University of Gondar, Gondar, Ethiopia; 4 Department of Clinical Midwifery, School of Midwifery, College of Medicine and Health Sciences, University of Gondar, Gondar, Ethiopia; 5 Department of Internal medicine, School of Medicine, University of Gondar Comprehensive Specialized Hospital, University of Gondar, Gondar, Ethiopia; 6 Department of Pharmaceutical Chemistry, School of Pharmacy, College of Medicine and Health Sciences, University of Gondar, Gondar, Ethiopia; 7 Department of Anesthesia, School of Medicine, College of Medicine and Health Sciences, University of Gondar, Gondar, Ethiopia; 8 Department of Epidemiology and Biostatistics, Institute of Public Health, College of Medicine and Health Sciences, University of Gondar, Gondar, Ethiopia; OAUTHC: Obafemi Awolowo University Teaching Hospital Complex, NIGERIA

## Abstract

**Objective:**

This study aimed to investigate long-acting reversible and permanent contraceptives (LARPCs) utilization and its associated factors among married women who desire no more children in Ethiopia.

**Methods:**

Secondary datasets from the 2016 Ethiopian Demographic and Health Survey was used for the study. A total weighted sample of 3,756 married or in union reproductive age women who desire no more children were included in the analysis. Data was cleaned, weighted, and analyzed using STATA Version 14 software. A multi-level logistic regression analysis was conducted to consider the hierarchal nature of the demographic and health survey data. In a multivariable multilevel logistic regression model, an adjusted odds ratio (AOR) with a corresponding 95% confidence interval (CI) and p value <0.05 was used to declare the significant associated factors of LARPCs utilization.

**Results:**

The overall utilization of LARPCs among married women who desire no more children was 12% [95%CI: 10.99, 13.07]. In the multivariable multilevel analysis; being female household heads [AOR = 0.60; 95%CI: 0.40, 0.92], husband primary level of education [AOR = 1.57; 95%CI: 1.18, 2.07], employed women [AO R = 1.34; 95%CI: 1.04, 1.74], women from middle wealth index [AOR = 1.45; 95%CI: 1.02, 2.07], women who visited health facility in the last 12 months [AOR = 0.69; 95%CI: 0.54, 0.88], women residing in small peripherals [AOR = 0.20, 95%CI: 0.05, 0.82], and women from communities with low poverty [AOR = 2.25, 95%CI: 1.26, 3.99] were significantly associated with LARPCs utilization.

**Conclusion:**

In Ethiopia, LARPCs utilization among married women who desire no more children was very low. Both individual and community-level factors were significantly associated with LARPCs utilization. Thus, individual and community-level interventions that encourage husband education, maternal occupation, and giving special attention for women who live in small peripheral areas and female-headed households are better.

## Introduction

Unintended pregnancies continue to be a major global public health concern posing the women for morbidity and mortality mainly due to unsafe abortion, as well as economic, social, and health crises for individuals and the community at large [1–4]. Worldwide, nearly half of pregnancies are unintended, with an estimated 121 million unintended pregnancies occurring each year; of these, 60% end in abortions, and 45% of these are unsafe. Which causes around 5 to 13% of maternal deaths [5]. Countries from low- and middle-income levels suffer the high burden, in which 74 million of the unintended pregnancies each year are from these countries, and 25 million and 47,000 of these pregnancies will end up with unsafe abortion and maternal mortality, respectively [1]. The burden varies from region to region, and the pooled prevalence of unintended pregnancy in these low- and middle-income countries is 26.6%, ranging from 19.25% in Egypt to 61.71% in Boliva [6]. Similarly, the prevalence of unintended pregnancy in Ethiopia is estimated to be 26.6–29.7% [7–9]. Unintended pregnancy is associated with negative socioeconomic and health outcomes for both women and children, families, and the community [4,10,11].

Ensuring availability and accessibility of reproductive health services, including family planning, is not only a matter of human rights, but it is also a key strategy for improving maternal and child health [12,13]. Globally, contraceptive use averted around 44% of maternal deaths [12]. By using modern contraception, women and girls can prevent unintended pregnancies, unwanted births, and higher-risk pregnancies, all of which contribute directly to maternal morbidities and mortality [14,15]. In spite of this, around 257 million women who desire to avoid getting pregnant do not use safe and modern methods of contraception worldwide [5].

Long Acting Reversible and Permanent Contraceptives (LARPCs), which include implants, intrauterine contraceptive devices (IUD), and sterilization can prevent pregnancy from 3 years to life-long, which are better choices to prevent unintended pregnancy due to their high efficacy and better safety, yet are little used in Ethiopia [16–18]. Evidence revealed that women who are using short-acting contraception are 21 times more likely to have an unplanned pregnancy than women using long-acting reversible contraceptives (LARC) [19]. Thus, these safe and more effective, with very low failure rate, contraceptives are the ideal form of methods for women who want to limit their childbearing [20,21]. Despite of the effectiveness of LARPCs to prevent unintended pregnancy, the actual utilization of LARPCs among women who desire no more children in sub-Saharan Africa (SSA) is 7.5% [22]. Similarly, regardless of the increasing utilization of modern contraceptives in Ethiopia, LARPCs uptake is still low compared to the short-acting contraceptive method [23].

Studies conducted on LARPCs utilization showed that maternal age [22,24], place of residence [24,25], wealth status [25], media exposure [22], maternal education status [22,24–26], husband education status [22,24], women participating in making their own health care decisions [22], household wealth index [22,25], knowledge about LARPCs [26], and number of living children [24] were statistically significant associated factors of LARPCs utilization.

Despite significant advancements in contraceptive technologies, unintended pregnancies remain a substantial public health issue in Ethiopia. To our knowledge, no study had been conducted at the national level in Ethiopia on the utilization of LARPCs among women who desire no more children, and community-level factors that might affect LARPCs utilization were largely overlooked. Therefore, this study seeks to use a nationally representative data to determine the prevalence and factors associated with LARPCs utilization among married women who desire no more children. The results of this study will help policymakers in the implementation of interventions that decrease unintended pregnancy and its consequences and will contribute to the promotion of maternal health in Ethiopia. Besides, identifying the associated factors affecting the use of LARPCs among married women who desire no more children is crucial to prevent unwanted pregnancy.

## Materials and methods

### Study design, setting and period

In this study, a community-based cross-sectional study design was done based on the Ethiopian Demographic and Health Survey (EDHS) 2016 data. The survey was conducted from January 18/2016, to June 27/2016. Ethiopia is found in the horn of Africa and is the second-most populous country in Africa. Ethiopia has two administrative cities (Addis Ababa and Dire Daw) and eleven regional states (Afar, Amhara, Benishangul-Gumuz, Gambella, Harari, Oromia, Somali, Southern Nations Nationalities and People’s Region (SNNPR), Tigray, Sidama, and Southwest) but during the data collection period, the new region Sidama and Southwest regions were under SNNPR. Each region are divided into zones, and the zones are into administrative units called Woreda. Each Woreda is furtherly divided into Kebeles, which are the smallest administrative entities in Ethiopia. Besides, Kebele is subdivided into census enumeration areas (EAs) [27].

### Data source, study population and sampling procedure

**Fig 1:**
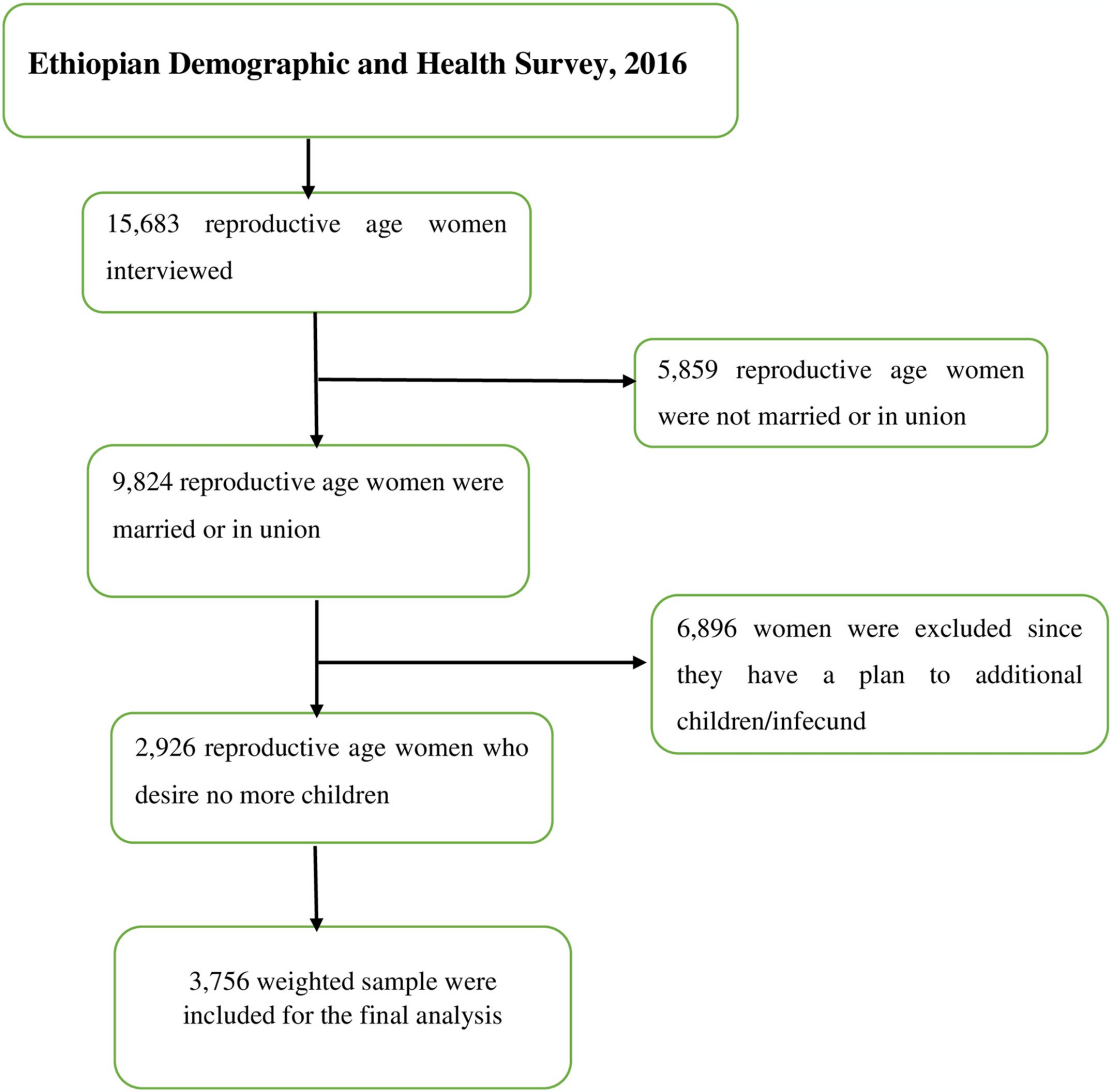
Schematic presentation shows the data extraction and sampling procedure.

The present study’s source of data was based on the 2016 EDHS. The 2016 EDHS was the fourth survey conducted in the country. All reproductive-age (15–49 years) married women who desire no more children in the selected enumeration areas (EAs) were the study population. Data were collected using a structured, interviewer-administered questionnaire every five years. It consists of various datasets, including women, children, birth, men, and household. In this particular study, we used the women’s datasets (IR file). To restore the survey’s representativeness, the data were weighted using an individual sampling weight for women (v005) divided by 1,000,000 before any statistical analysis. A total weighted sample of 3756 married women who desire no more children were included in the analysis (Fig 1).

To select the study participants, the EDHS used a two-stage stratified cluster sampling technique using the 2007 Population and Housing Census as a sampling frame. Stratification was done by separating each region into rural and urban areas. In the stage one, 645 EAs were selected. Among this, 443 were from rural areas. On average, 28 households per each cluster were selected with an equal probability systematically selection in the second stage. Further information about the data/survey has been included in the 2016 EDHS report [28].

### Variables of the study

#### Outcome variable

In this study, the outcome variable was LARPCs utilization, which was a binary outcome variable coded as "1" if a woman utilized LARPCs (IUD, implant, and sterilization) and "0" if a woman did not utilize LARPCs.

#### Independent variables

Both individual and community-level independent variables were considered in this study. The individual-level factors included were maternal age, sex of household head, maternal education status, husband education status, maternal occupation status, husband occupation status, household wealth index, number of alive children, visiting a health facility in the past 12 months, women participating in making their own health care decisions, knowledge of LARPCs, and media exposure. In this study, wealth index was categorized as; poor “if woman was in poorer and poorest household”, middle “if woman was in middle household”, and rich “if woman was in richer and richest household” [29,30]. Media exposure was determined from three variables, such as the frequency of reading a newspaper or magazine, listening to the radio, and watching TV, and categorized into exposed (if they had been exposed to either of the three) and unexposed (if they had no exposure to either of the three) [31,32]. Six variables, such as place of residence, region, community-level women’s education, community-level media exposure, community-level poverty, and distance from the health facility were considered as community-level factors. Region was recoded as Metropolis (such as Addis Ababa, Harari, and Dire Dawa), larger central regions (Tigray, Amhara, Oromia, and SNNPRs), and small peripherals (such as Afar, Somali, Benishangul, and Gambella) [33].

From community level variables, place of residence, region, and distance to health facility were a non-aggregate while community women’s education, community media exposure, and community poverty were created by aggregating individual-level variables at the cluster level. The community-level poverty variable was measured by the proportion of women in the poorer and poorest wealth index, and it was dichotomized as low (communities with <50% women had poorer and poorest wealth quintiles) and high (communities with ≥50% of women had poorer and poorest wealth quintiles) based on median value. Community women’s education was measured by the proportion of women who had at least a primary level of education in a cluster. Then, based on the median value, it was categorized as low (communities with <50% of women had at least a primary level of education) and high (communities with ≥ 50% of women had at least a primary level of education). Community media exposure was also determined by the proportion of women who have been exposed to at least one media (television, radio, or newspaper) in a cluster and classified as low (communities with <50% of women exposed) and high (communities with ≥50% of women exposed) according to the median value [34,35].

### Data management and statically analysis

Outcome and independent variables were extracted in the EDHS, and the data were cleaned, recoded and further analysis was done using STATA version 14 software. Before conducting any statistical analysis, EDHS data were weighted using sampling weight (v005) to ensure the survey representativeness and to draw valid inferences.

### Model building

Since EDHS data has a hierarchical structure with individuals nested within clusters, we anticipate that women within the same cluster may be more similar to one another than women in another cluster. As a result, we conducted a multilevel logistic regression analysis. In the multilevel logistic regression analysis, four models were fitted. Model I (the null model) contains only the outcome variable. Model II includes the outcome variable with individual-level variables, and Model III contains the outcome variable with community-level variables. In the final model (model IV), both individual and community-level variables with LARPCs utilization were fitted simultaneously.

### Parameter estimation method

The fixed effects (a measure of association) were used to determine the association between the likelihood of LARPCs utilization among married women who desire no more children and independent variables at both community and individual levels. Before multivariable multilevel logistic regression analysis, bivariable regression analysis was done and those variables with a p-value of ≤ 0.2 were considered for multivariable multilevel regression analysis. In the multivariable analysis, adjusted odds ratios (AOR) and 95% confidence intervals (CI) with a p value <0.05 were computed to declare the associations between dependent and independent variables. Multicollinearity was checked using the variance inflation factor (VIF) test and there was no multicollinearity between independent variables since the mean VIF was 1.8.

Regarding random effects analysis, which is used to measure the variation of LARPC utilization across communities or clusters, were assessed using the Intra-cluster Correlation Coefficient (ICC), Median Odds Ratio (MOR), and Proportional Change in Variance (PCV). The ICC shows the variation of LARPC utilization between clusters and it is computed by $ICC=\frac{\text{VA}}{\text{VA}+3.29}*100\%$ where VA = cluster level variance. Median odds ratio (MOR) is defined as the median value of the odds ratio between the area at the lowest risk and at the highest risk of LARPCs utilization when randomly selecting two women from two clusters. It is calculated as;

$$\text{MOR}=e^{0.95\sqrt{VA}},$$

where VA is the cluster level variance.

The PCV also reveals the total variation of LARPCs utilization explained by the final model (a model with both individual-level and community-level variables) relative to the null model (a model only with dependent variables). $PCV=\frac{Vnull-VA}{V\;null}*100\%$, where Vnull is variance of the initial model, and VA = cluster level variance of the next model [36,37].

### Ethical consideration

Since the study was a secondary data analysis of the 2016 EDHS data, ethical approval and participant consent were not necessary for this particular study. When the EDHS was conducted, informed consent was obtained. There were no names of individuals or household addresses in the data file. There is no experimentation in this study. The EDHS data is available to the public by request in different formats from Measure DHS at http://goo.gl/ny8T6X. We obtained permission to download the dataset in STATA format.

## Results

### Sociodemographic characteristics of study participants

A total weighted sample of 3756 married women aged 15–49 who desired no more children were included in this study. Of these, 88.88% were from male-headed households, and 71.62% of women had no formal education. The median age of women was 35 (IQR ± 11) years. Nearly two third (63.3%) of the respondents had no media exposure, and 39.12% of women were from poor households. Regarding women participating in making their own health care decisions, about 82.15% of the women participated in making their own health care decisions (Table 1).

**Table 1:** Background characteristics of study participants in a study of LARPCs utilization and its associated factors among married women who desire no more children in Ethiopia: Based on 2016 EDHS.

Variables	Weighted frequency	Percentage (100%)
**Age**		
15–24	264	7.02
25–34	1,360	36.20
≥ 35	2,132	56.77
**Sex of household head**		
Male	3,338	88.88
Female	418	11.12
**Maternal education status**		
Have no formal education	2,690	71.62
Primary education	884	23.53
Secondary and above	182	4.85
**Husband/partner’s education**		
Have no formal education	1,917	51.04
Primary education	1,415	37.68
Secondary and above	424	11.28
**Maternal occupation status**		
Unemployed	1,888	50.26
Employee	1,868	49.74
**Husband/partner occupation status**		
Unemployed	309	8.22
Employee	3,447	91.78
**Wealth index**		
Poor	1,469	39.12
Middle	776	20.66
Rich	1,511	40.22
**Number of alive children**		
No children	27	0.72
1–2	472	12.58
3–4	1,066	28.37
Five and above	2,191	58.33
**Media exposure**		
Unexposed	2,399	63.87
Exposed	1,357	36.13
**Visiting health facility in the last 12 months**		
No	1,994	53.09
Yes	1,762	46.91
**Women participating in making their own health care decisions**		
Yes	3,085	82.15
No	671	17.85
**Knowledge of LARPCs**		
Yes	579	15.43
No	3,177	84.57

### Community-level characteristics of the study participants

The majority (86.90%) of the respondents were rural residents. Almost half (48.81%) of women were from a community with low poverty, and 57.31% of the women reported perceived distance to visit health facilities as a big problem (Table 2).

**Table 2:** Community level characteristics of study participants: Based on EDHS 2016.

Variables	Weighted frequency	Percentage (100%)
**Residency**		
Urban	492	13.10
Rural	3,264	86.90
**Region**		
Metropolis	122	3.25
Large centrals	3,548	94.45
Small peripherals	86	2.30
**Distance to health facility**		
Not a big problem	1,604	42.69
Big problem	2,152	57.31
**Community level media exposure**		
Low	1,679	44.69
High	2,077	55.31
**Community level of poverty**		
Low	1,833	48.81
High	1,923	51.19
**Community level of women education**		
Low	2,257	60.10
High	1,499	39.90

### Long-acting reversible and permanent contraceptives utilization among married women who desire no more children in Ethiopia

**Fig 2:**
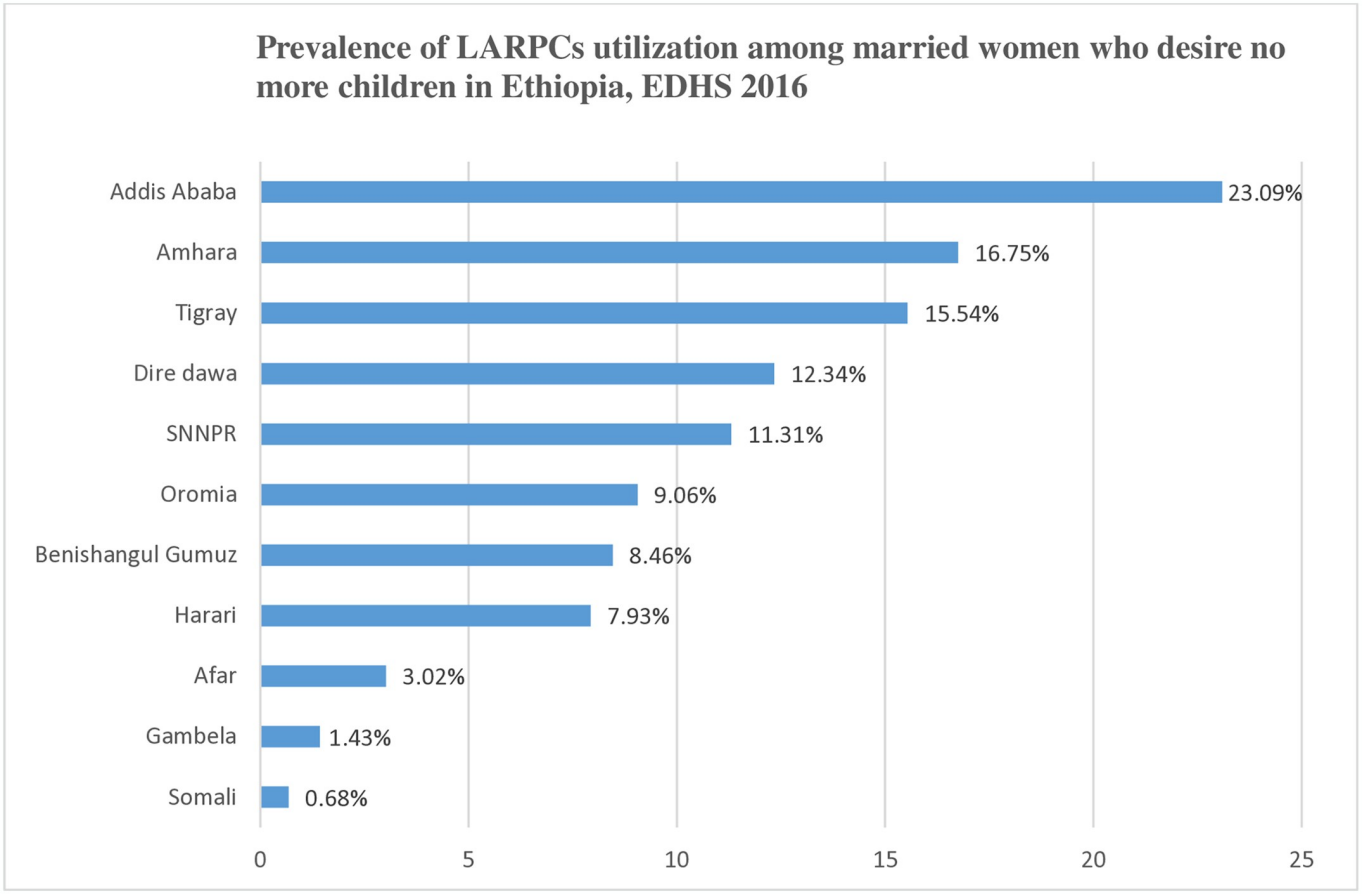
Regional LARPCs utilization among married women who desire no more children in Ethiopia, EDHS 2016.

In this study, the overall utilization of LARPCs among married women who desire no more children was 12% [95%CI: 10.99, 13.07]. The highest level of LARPCs utilization was seen in the Addis Ababa region (23.09%), and the lowest level of LARPCs utilization was seen from Somali region (0.68%) (Fig 2).

### Model comparison and random effect analysis

As shown in Table 3, model comparison/fitness was done using the Log-likelihood and deviance tests, and then the final model (model III) was the best-fitted model since it had the highest log likelihood (−1172) and the lowest deviance value (2344). The ICC value in the null model was 43% indicated that 43% of the total variability of LARPC utilization was due to differences between clusters, whereas the remaining unexplained 57% was attributable to individual differences. The null model also had the highest MOR value (4.41), which indicates that if we randomly select two women from different clusters, a woman from a cluster with higher LARPC utilization was 4.41 times more likely to utilize the LARPC than a woman from a cluster with lower LARPC utilization. Besides, PCV was 23% in the final model (model III), which indicated that about 23% of the variation in LARPC utilization among women who desire no more children was explained by combined factors at both the individual and community level.

**Table 3:** Multilevel logistic regression analysis of individual and community level factors associated with LARPCs utilization among married women who desire no more children in Ethiopia: Based on 2016 EDHS.

Variables	Model I	Model II	Model III	Model IV
	Null model	AOR (95%CI)	AOR (95%CI)	AOR (95%CI)
**Age**				
15–24		1		1
25–34		1.52 (0.87, 2.66)		1.48 (0.84, 2.58)
≥ 35		1.39 (0.81, 2.42)		1.36 (0.78, 2.35)
**Sex of household head**				
Male		1		1
Female		0.63 (0.42, 0.96)		0.60 (0.40, 0.92)*
**Maternal education status**				
Have no formal education		1		1
Primary education		0.78 (0.57, 1.08)		0.73 (0.52, 1.01)
Secondary and above		1.18 (0.60, 2.29)		0.86 (0.43, 1.73)
**Husband/partner’s education status**				
Have no formal education		1		1
Primary education		1.59 (1.21, 2.11)		1.57 (1.18, 2.07)**
Secondary and above		1.19 (0.72, 2.00)		1.09 (0.65, 1.83)
**Maternal occupation status**				
Unemployed		1		1
Employee		1.36 (1.06, 1.76)		1.34 (1.04, 1.74)*
**Wealth index**				
Poor		1		1
Middle		1.70 (1.21, 2.40)		1.45 (1.02, 2.07)*
Riche		1.27 (0.91, 1.79)		0.95 (0.66, 1.37)
**Visiting health facility in the last 12 months**				
No		1		1
Yes		0.71 (0.55, 0.91)		0.69 (0.54, 0.88)**
**Women participating in making their own heath care decisions**				
Yes		1.28 (0.90, 1.83)		1.25 (0.88, 1.79)
No		1		1
**Community level variables**				
**Residency**				
Urban			0.89 (0.43, 1.86)	1.15 (0.54, 2.43)
Rural			1	1
**Region**				
Metropolis			1	1
Large centrals			0.61 (0.26, 1.43)	0.56 (0.24, 1.34)
Small peripherals			0.21 (0.05, 0.81)	0.20 (0.05, 0.82)*
**Community level media exposure**				
Low			1	1
High			1.13 (0.67, 1.92)	1.18 (0.69, 2.01)
**Community level poverty**				
Low			2.36 (1.36, 4.08)	2.25 (1.26, 3.99)**
High			1	1
**Community level women education**				
Low			1	1
High			1.12 (0.67, 1.88)	1.21 (0.71, 2.07)
**Random effect**				
VA	2.44	2.36	2.29	1.87
ICC	0.43	0.42	0.41	0.36
MOR	4.41	4.30	4.21	3.66
PCV	Reff	0.03	0.06	0.23
**Model comparison**				
Deviance (-2LLR)	2418	2370	2390	2344

Note;

* = P-value < 0.05,

** = Pvalue < 0.01.

ICC = Inter-cluster corrolation cofficent, MOR = Median odds ratio, PCV = proportional change in variance, AOR = adjusted odds ratio; CI = confidence interval.

### Factors associated with LARPCs utilization among married women who desire no more in Ethiopia

In multivariable multilevel logistic regression analysis, variables such as sex of household head, husband education status, women occupation status, wealth status, visiting health facility in the last 12 months, community level poverty, and region were significantly associated with LARPCs utilization.

From individual level variables, women from female head households had 40% [AOR = 0.60; 95%CI: 0.40, 0.92] less likely to utilize LARPCs than women from male head households. Women whose husbands had a primary level of education had 1.57 times [AOR = 1.57; 95%CI: 1.18, 2.07] higher odds of LARPCs utilization than women whose husbands did not have formal education. The odds of LARPCs utilization among women who had an occupation were 1.34 times [AOR = 1.34; 95%CI: 1.04, 1.74] higher than women who did not have an occupation. Women from a household with middle wealth status had 1.45 times [AOR = 1.45; 95%CI: 1.02, 2.07] higher odds of LARPCs utilization than women from a household with poor wealth status. The odds of LARPCs utilization among women who visited health facility in the last 12 months were 31% [AOR = 0.69; 95%CI: 0.54, 0.88] lower as compared to their counterparts.

Regarding community level factors, the odds of LARPCs utilization among women residing in small peripheral areas decreased by 80% [AOR = 0.20; 95%CI: 0.05, 0.82] as compared to women residing in metropolis areas. Women from communities with low poverty were 2.25 [AOR = 0.44; 95%CI: 0.25, 0.79] times more likely to utilize LARPCs as compared with those from communities with high poverty (Table 3).

## Discussion

This study attempted to investigate the prevalence and associated factors of LARPCs utilization among married women who desire no more children in Ethiopia based on the nationally representative EDHS data. In this study, the prevalence of LARPCs utilization among women who desire no more children was found to be 12%. This study finding is lower than studies done in Gondar, Ethiopia [26] and Indonesia [24]. The possible justification for this discrepancy might be the study setting difference. In previous studies, the study was done at the health facility level among women who desired no more children and came for family planning services, while our study was conducted at the community level. Moreover, the majority of study participants in the study mentioned above were urban residents, who might have better access to information about contraceptive methods and could easily access the services than those in rural areas, where health facilities are somewhat far away in the Ethiopian context [38].

On the other hand, the finding of this study is higher than a study done in sub-Saharan Africa [22]. The possible explanation for this discrepancy might be that the expansion of the health extension program in Ethiopia might have played a role in awareness creation and family planning service utilization [39,40]. Besides, the discrepancy of this finding could be due to socio-demographic and cultural deference. In this study, the majority of the study participants decided on their health care. Therefore, they can able to utilize LARPCs and increase the prevalence of utilization.

In the multilevel logistic regression analysis; sex of household head, husband education status, maternal occupation status, wealth status, visiting health facility in the last 12 months, community level poverty, and region were significantly associated with LARPCs utilization. Consistent with different studies conducted in Ethiopia [41,42] and Lesotho [43], female household heads had lower odds of LARPCs utilization as compared with households whose head was male. The possible explanation might be that a husband may be away from home for different reasons, and women may have less sexual intercourse. As a result, the woman may not use contraceptives or may use a short-acting contraceptive method. This may be due to the fact that awareness towards unwanted pregnancy, its consequences, and health service-seeking behaviors for any health problems might be lower in female-headed households.

In this study, husband education was a significant factor of LARPCs utilization. Women whose husbands had a primary level of education had higher odds of LARPCs utilization as compared to women whose husbands did not have formal education. It was consistent with studies reported in SSA [22] and Indonesia [24]. The potential explanation may be that husband education plays a crucial role in enhancing the use of maternal health care services like contraceptives and increasing awareness of unintended pregnancy consequences. Besides, husbands education positively increases their wives health-seeking behaviors [44].

Congruent with studies done in Ethiopia [45,46] and East Africa [47], women having occupation in this study showed higher odds of LARPCs utilization when compared with their counterparts. The possible justification might be the reason that women who have occupation and have their own income may have better social media and health facility access that enhances LARPCs utilization. Moreover, these women’s occupations may reflect their educational status, which will contribute to LARPCs service utilization. Besides, they may not miss LARPCs as they are on duty. They might also have a higher chance of accessing and utilizing different social media.

The odds of LARPCs utilization were higher among women from a household with middle wealth status and low community poverty compared to those from a household with poor wealth status and high community poverty. This finding is supported by studies conducted in Ethiopia [48], Ghana [49], and SSA [22]. The possible explanation is that women in middle household wealth status and low community level poverty may have greater access to media exposure, education, and health services including the LARPCs [50]. This increases women’s awareness regarding the purpose of LARPCs utilization and the adverse effects of an unwanted pregnancy. They may also have greater autonomy and decision-making power within their relationships, allowing them to make informed choices about their reproductive health.

Our study also revealed that visits to health facility in the last 12 months were significantly associated with LARPCs utilization. The odds of LARPCs utilization among women who visited health facility in the last 12 months were 31% lower as compared to their counterparts. The possible reason might be that women who have visited health facility in the last 12 months may have bad prior experience related to privacy or confidentiality and a long waiting time for their last visit. These might lead to a decreased LARPCs utilization. Therefore, providing comprehensive and unbiased information about contraceptive options, including the LARPC method, during their health facility visits is crucial for promoting informed decision-making and increasing utilization rates among all women, regardless of their healthcare-seeking behavior.

This study found that region was an important community level associated factor for LARPCs utilization. Women from small peripheral regions were less likely to utilize LARPCs as compared to women from metropolitan areas. This is congruent with studies done in Ethiopia at different period [51,52]. The possible explanation for this might be that these areas are more of a pastoralist place, where people do not own a permanent place or residence, and therefore women are facing problems in accessing maternal health care services due to health facilities that are not available and accessible as compared with those from cities [53,54]. Besides, women in pastoral regions have poor access to education, and because of this, women may not have awareness about contraceptive services, unwanted pregnancy, and its consequences, which would lead to a decrease in LARPCs utilization [55].

Our study had strength. Since it was based on nationally representative data with large sample size, the study has the potential to give insight for program planners and policymakers into how to design appropriate intervention strategies at both regional and national levels. The other strength of this study was that, to accommodate the hierarchical nature of the data, we used an appropriate statistical approach (multilevel analysis). Furthermore, this study revealed evidence that LARPC service utilization in Ethiopia is not only affected by women’s individual-level characteristics but also by the community’s-level factors. Despite the aforementioned strengths, this study had its own limitations. The cross-sectional nature of the data does not allow for a cause-and-effect relationship between the outcome and independent variables. Besides, the EDHS data relied on women’s verbal autopsy, and therefore there is a possibility of social desirability bias.

## Conclusion

This study showed that LARPCs utilization among married women who desire no more children in Ethiopia remains unacceptably low. The study is done using an advanced model to take into account the clustering effect (mixed-effect logistic regression) in order to get a reliable standard error and estimate. Sex of household head, husband education, maternal occupation, wealth status, visited health facility in the last 12 months, region, and community level poverty were significantly associated with LARPCs utilization. Thus, governmental and non-governmental organizations and policymakers could strengthen the effort towards maternal and reproductive health services, specifically for small peripheral residents and women who live in female-headed households. It is better to strengthen husband education and maternal occupation. Besides, we recommend researchers to explore the reason why married women who desire no more children do not utilize LARPCs using a qualitative study.

## Abbreviations

AOR: Adjusted Odds Ratio
CI: Confidence Interval
DHS: Demographic Health Survey
EA: Enumeration Area
EDHS: Ethiopian Demographic Health Survey
ICC: Intra-cluster Correlation Coefficient
IUD: Intrauterine Contraceptive Devices
LARPC: Long Acting Reversible and Permanent Contraceptive
LLR: Log-likelihood Ratio
MOR: Median Odds Ratio
PCV: Proportional Change in Variance
SNNPR: Southern Nations Nationalities and People’s Region
VIF: Variance Inflation Factor
